# Response of the Chinese Soft-Shelled Turtle to Acute Heat Stress: Insights From the Systematic Antioxidant Defense

**DOI:** 10.3389/fphys.2019.00710

**Published:** 2019-06-06

**Authors:** Wenyi Zhang, Bojian Chen, Cuijuan Niu, Lin Yuan, Hui Jia, Kenneth B. Storey

**Affiliations:** ^1^Key Laboratory for Biodiversity Science and Ecological Engineering, Ministry of Education, College of Life Sciences, Beijing Normal University, Beijing, China; ^2^State Key Laboratory of Marine Environmental Science, College of Ocean and Earth Sciences, Xiamen University, Xiamen, China; ^3^College of Environmental Science and Engineering, Tongji University, Shanghai, China; ^4^Department of Biology and Institute of Biochemistry, Carleton University, Ottawa, ON, Canada

**Keywords:** acute temperature elevation, antioxidant defense, ascorbic acid, freshwater turtle, glutathione

## Abstract

Understanding the responses of animals to acute heat stress can help to reveal and predict the effect of more frequent extreme hot weather episodes on animal populations and ecosystems in the content of global climate change. Antioxidant defenses can help to protect animals against oxidative stress caused by intense temperature variation. In the present study, systematic antioxidant responses to acute heat stress (Δ15°C and maintained for 12 h) and subsequent recovery were assessed by evaluating gene transcript levels and relative enzyme activities in tissues of *Pelodiscus sinensis*, a subtropical freshwater turtle. Targets included nuclear factor erythroid 2-related factor 2 (Nrf2, the upstream transcription factor), antioxidant enzymes, and the glutathione (GSH) and ascorbic acid (AA) systems. Results showed three main patterns of expression change among antioxidant genes: (1) gene expression of Mn-superoxide dismutase (Mn-SOD), glutathione peroxidase 4 (GPx 4), and catalase (CAT) increased in response to heat stress or recovery in the liver; (2) transcripts of most genes did not change in brain, liver, and kidney of *P. sinensis*; and (3) expression of several GST isoforms were affected by heat stress or recovery in brain and kidney. However, relative enzyme activities involved in antioxidant defense were little affected by acute heat stress and recovery, indicating a relatively conservative antioxidant response in *P. sinensis*. Furthermore, results for malondialdehyde (MDA) levels indicated that acute heat stress and recovery did not cause a net increase in oxidative damage in turtle tissues and, in particular, MDA levels in spleen decreased along with increased splenic ascorbic acid concentration. Overall, the present study revealed a conservative antioxidant response in *P. sinensis*, which may be indicative of a high basal stress tolerance and relate with adaptation to climate change in freshwater turtles.

## Introduction

An increase in the intensity and frequency of extreme weather is predicted to occur as the result of global climate change (IPCC, 2014). Extreme weather can acutely and significantly affect behavior, growth, and physiology of animals and in some cases cause mortality (Mckechnie and Wolf, 2010; Tinsley et al., 2015). Many species have adapted over evolutionary time to seasonal changes in weather (Mousseau and Roff, 1989; Hoffmann and Sgrò, 2011). However, many extreme weather events that are associated with rapid temperature elevation or drastic temperature variation can directly affect survival and physiological performance, such as energy metabolism or immunity, of animals (Gillooly et al., 2001; Zhang et al., 2015). Therefore, it is crucial to understand the effects of acute heat stress caused by extreme hot weather on animals.

There are typically three options for animals responding to changing environmental conditions: (1) move to a different geographic habitat or range (Hahn et al., 2008), (2) physiological responses/adaptations (Breuner and Hahn, 2003), or (3) death. Extreme weather change usually happens very quickly with no prior signaling and, thus, a rapid physiological response generally takes priority, especially in those animals with low mobility. Antioxidant defense, which includes antioxidant enzymes and low molecular weight oxyradical scavengers, is an important physiological component of the stress response (Hermes-Lima et al., 2001). Antioxidant defenses work to clear overproduction of reactive oxygen species (ROS), which are often by-products of disrupted mitochondrial respiration and can be induced by multiple environmental stresses, including temperature variation, hypoxia, UV radiation or exposures to toxic chemicals or metals (Leveelahti et al., 2014; Szekeres et al., 2014; Ibrahim, 2015). Antioxidant enzymes, including superoxide dismutase (SOD), catalase (CAT), and glutathione peroxidase (GPx), catalyze reactions to destroy ROS (Hermes-Lima et al., 2001). In addition, low molecular weight scavengers, such as glutathione (GSH) or ascorbic acid (AA), also play important roles as reductants in eliminating ROS (Meister and Anderson, 1983; Rice et al., 1995). Insufficient antioxidant defenses result in accumulation of oxidative damage on biomacromolecules, including DNA, protein and lipid, that can lead to final death. Therefore, antioxidant responses to adverse stress can affect the survival of animals and be related to fitness (David et al., 2010; Nicola et al., 2011). Among ectothermic or endothermic vertebrates, numerous studies have revealed enhanced antioxidant defenses during or following environmental stress, indicating that antioxidant defenses are an essential part of stress tolerance and that they are induced in many situations as a preparation for oxidative stress (Lushchak and Bagnyukova, 2006; Schülke et al., 2012; Oliveira et al., 2018).

Turtles are important ectotherms in freshwater systems, where they can experience seasonal or episodic thermal stress. However, there is little information about antioxidant responses by freshwater turtles to acute heat stress. Studies have shown that strong antioxidant defenses benefit the stress tolerance of freshwater turtles when they exposed to environmental stresses, including acute temperature reduction, supercooling, freezing, or hypoxia (Churchill et al., 1997; Willmore and Storey, 1997a,b; Zhang et al., 2017b). Among these studies of freshwater turtles, a relatively conservative antioxidant response is often indicated by a mild mRNA transcript response and generally stable enzyme activities. However, several questions concerning antioxidant regulation in the heat stress response of freshwater turtles are unanswered. Stresses employed in previous studies, including low temperature, freezing or hypoxia, can be considered as being energy limited or causing metabolic inhibition. However, acute heat stress will clearly elevate the metabolic rate of ectotherms, with consequences for the antioxidant response that are unknown. Therefore, analysis of the systemic antioxidant response to acute heat stress is essential for understanding the stress response strategy of freshwater turtles and revealing their physiological capacity to respond to extreme hot weather.

In the present study, we examined the systematic antioxidant responses by the Chinese soft-shelled turtle, *Pelodiscus sinensis* to acute heat stress (Δ15°C in less than 5 min and maintained for 12 h) and following recovery. This species has previously been shown to have good cold tolerance as well as resilience (Zhang et al., 2017b, 2018). We measured changes in the expression levels of nuclear factor erythroid2-related factor 2 (Nrf2), a principal regulator of many antioxidant genes, the mRNA transcript responses by multiple antioxidant enzyme genes (e.g., SOD, CAT, and GPx) and the relative activities of antioxidant enzymes in the brain, liver, and kidney of *P. sinensis*. Changes in mRNA transcript and relative enzyme activities of GSH related enzymes, including glutathione synthetase (GS), glutathione reductase (GR), and glutathione-S-transferase (GST), were also measured in these three tissues as well as L-gulonolactone oxidase (GLO), the key enzyme that regulates AA synthase in the kidney. Levels of AA and the lipid peroxidation biomarker, malondialdehyde (MDA), were also quantified in brain, liver, spleen, kidney, and plasma of *P. sinensis.* MDA was selected because *P. sinensis* is rich in unsaturated fatty acid and MDA was sensitive to acute temperature variation in our previous studies (Huang et al., 2005; Zhang et al., 2017b; Chen et al., 2018). Based on the previous findings in antioxidant strategy of the turtles, we predicted the use of a conservative antioxidant response, composed of small changes in transcript levels and relatively stable enzyme activities, in *P. sinensis* in response to acute heat stress and recovery thereafter.

## Materials and Methods

### Ethics Statement

The experiments were conducted according to the standards of the Ethics and Animal Welfare Committee (EAWC) of Beijing Normal University (Approval No. CLS-EAW-2014-010).

### Animal Treatment

The optimum growth temperature for *P. sinensis* ranges from 25 to 32°C. When temperature drops below 15°C, the turtles reduce their physiological activities and prepare for hibernation (Zhang et al., 2017a; Chen et al., 2018); whereas when water temperature rises above 35°C, they enter torpor and die quickly (Niu et al., 1998). Periods of extreme hot weather normally occur in summer, during which the ambient temperature allows fully functional physiological performance of *P. sinensis*, but the higher end of thermal fluctuation may push the turtles beyond their survival limits. Hence in order to investigate the maximum antioxidant response in the turtle’s functional thermal range, we decided to set the acclimation temperature at 18°C, and the heat stress temperature at 33°C.

#### Acclimation

Juvenile turtles, *P. sinensis* (*n* = 48, mean = 107.1 ± 1.6 g) were purchased from a turtle hatchery (Yutian County, Hebei Province, China) and reared in the laboratory at 18 ± 1°C room and water temperatures for 3 weeks. The photoperiod was 12L/12D and the turtles were fed daily with commercial standard diets (Hebei Haitai Tech. Ltd., Shijiazhuang, China).

#### Acute Heat Stress

After acclimation, turtles were fasted for 48 h with water temperature maintained at 18 ± 1°C. A random selection of animals (*n* = 16) were then sacrificed as the control group. Water temperature was then raised acutely to 33°C in less than 5 min and maintained for 12 h. This 12 h time of high temperature stress was selected because it is a typical length of environmental stress applied in similar studies and is also probably the longest time that turtles would experience heat stress in their natural habitat considering diurnal patterns of environmental temperature variation. At the end of the 33°C heat treatment, 16 individuals were sacrificed randomly and defined as the heat stress group (*n* = 16, Δ15°C, 12 h). Then water temperature was dropped back to 18°C and maintained for 24 h to allow animals to recover. The remaining turtles, defined as the recovery group, were then sacrificed (*n* = 16). All turtles were sacrificed by quick decapitation (the typical mode of euthanasia in turtle studies) (Willmore and Storey, 1997a,b; Baker et al., 2007). Blood samples were collected from the neck-chest fracture section into pre-heparin sodium treated EP tubes and then centrifuged at 5500 g for 10 min. Plasma was removed into new tubes and then frozen. The brain, liver, kidney, and spleen were quickly excised, frozen in liquid nitrogen and transferred to -80°C for storage. For analysis of the antioxidant enzyme system, transcript levels and enzyme activities of SOD, CAT, and GPx were measured. For the GSH system and AA system, transcript levels and enzyme activities of GS, GR, GST, and GLO were measured. AA and MDA concentrations in tissues were also measured. Within each group, half of the turtles (*n* = 8) were employed to detect changes in the antioxidant enzyme system and the other half were used to assess the changes in GSH and AA systems.

### Total RNA Extraction and RT-PCR

The protocol was the same as in our previous studies (Chen et al., 2015; Zhang et al., 2017a). Total RNA was extracted from all tissues (30–100 mg) using a combination of Trizol reagent (Takara, Japan) and the Nucleospin RNA II kit (Macherey-Nagel, Germany) following the product manual. After RNA extraction, the quality and quantity of RNA (A260/280 > 1.9, concentration: 0.2–0.8 μg/μL) were determined using agarose gel electrophoresis and a NanoDrop 2000 spectrophotometer (Thermo, United States).

Reverse transcription was conducted with PrimerScript II first strand cDNA synthesis kit (Takara, Japan). A 2 μg amount of total RNA template was used for each 30 μL reaction. The cDNA was then diluted six times and stored at -20°C for use in real-time PCR.

### Real-Time PCR for Measurement of Gene Expression

Primer sequences, gene accession numbers and PCR efficiencies for real-time PCR are shown in Table 1. The PCR efficiency for each pair of primers was calculated in accordance with our previous study (Zhang et al., 2017a).

**Table 1:** Gene abbreviation, GenBank accession number, forward and reverse primer sequences, and real-time PCR efficiency for the target genes analyzed.

Gene abbreviation	Accession number	Forward primer	Reverse primer	PCR efficiency
*EF-1α*	NM_001286922.1	CCATCGTTGACATGGTCCCA	ACTTTGTGACCTTGCCAGCT	1.98 ± 0.04
*GAPDH*	NM_001286927.1	TTCATGGCACTGTCAAGGCT	GGTTGACGCCCATCACAAAC	1.91 ± 0.01
*Nrf2*	JX470526	GCAGCATCTTCCTTGTTCCTAAA	AGTTAGCTTCCTTGCCTGTCAAA	1.99 ± 0.03
*Cu/ZnSOD*	JX470524	TGCAGGTGCTCACTTCAATCC	CAACATGCCTCTCTTGATCTTGTG	2.05 ± 0.02
*MnSOD*	JX470525	GCCATCAAGCGTGATTTCG	CTGATACTGCTGTCAGCTTCTCCTT	1.96 ± 0.02
*CAT*	JX452102	GCAGCGCTTCAATAGTGCAA	GTTCATCTTCTTTCAGCACTTTGG	2.06 ± 0.01
*GPx1*	KC357250	GCTTCCCGTGCAACCAGTT	CGGCCGCACGTACTTGA	2.04 ± 0.03
*GPx3*	JX470527	AACCAGTTTGGCAAGCAAGAG	CGGGCCGGACGTATTTC	2.04 ± 0.00
*GPx4*	JX470528	GCCTCGTCTGCATCATCGT	GTGTAGTTCACGGCCGTCTTG	1.93 ± 0.01
*GS1*	XM_006116645.1	TTCCTGGAACGTGCTCTTG	CCAAACTTCCCTTCGGAAA	1.98 ± 0.02
*GS2*	XM_006116646.1	AAGCACCCTCTTTGAGCAAG	CCCAAGAAGACCGTCTGCA	1.99 ± 0.02
*GR*	XM_006125372.1	ACGTTGACTGTCTGCTGTGG	TGCAACTGGAGTCAGGAGTG	1.97 ± 0.01
*GST1*	XM_006130271.1	AGATACAGCGACGTTTGGCA	GCCCCAACGAAGATCCTGAA	1.96 ± 0.02
*GST3*	XM_006139372.1	ACCCTGAACACGGCCATATC	AGTATGAGTGCCGCTGAACC	1.94 ± 0.01
*GSTK1*	XM_006111374.1	ACAGCCGTGGATATGACACA	TCAGGCGATTCTTCACCTCT	1.98 ± 0.01
*GSTP1*	XM_006110953.1	ACGGAAACGTAACCCTGTACC	TAGATGAGCTGGGCGTATTTG	1.91 ± 0.01
*GSTC*	XM_006119312.1	TGGCAACTGACATGGTGATT	CCAGCCCCATACAGTGTTTT	1.97 ± 0.02
*GSTZ1_X1*	XM_006134809.1	ACTGGCAGCTTTAAGAAACCT	TCACTAAGGCACCACTCATCT	1.95 ± 0.01
*GSTZ1_X2*	XM_006134810.1	CCACCCCAGCTGAGTGAAAA	AAGAACCGGCTTTCCAGAGG	2.01 ± 0.03
*GLO*	HQ619721.1	TATCAGGACCACACCGACAA	AGAGCAGGAACTCCAGCAGA	2.04 ± 0.01


Real-time PCR was conducted on a 7500 real-time PCR system (Applied Biosystems, United States). The reaction condition included 10 μL of 2 × SYBR Green PCR Master Mix (Applied Biosystems, United States), 3 μL cDNA template, 20 μM each of forward and reverse primers and 6.5 μL Milli-Q H_2_O. Elongation factor 1 alpha 1 (EF-1α) was selected as the control gene for brain and liver whereas glyceraldehyde-3-phosphate dehydrogenase (GAPDH) was selected for kidney. The choice of different control genes for different tissues was based on prior testing of several putative control genes that revealed genes whose expression of (EF-1α or GAPDH) remained constant in a given tissue across all three experimental conditions. The relative expression of each gene was calculated in accordance with the 2^-ΔΔCT^ method (Schmittgen and Livak, 2008).

### Enzyme, Protein, and AA Assays

All enzyme activities were measured using Diagnostic Reagent Kits (Nanjing Jiancheng, China) except for GPx and GLO. The former was measured using a kit from Beyotime company (China), and the latter was determined using high-performance liquid chromatography (HPLC). Tissue samples were homogenized in a ninefold volume of ice-cold physiological saline (0.68% NaCl) and then activities were measured according to product manuals. Detailed methods for activity assays were descripted in our previous studies (Zhang et al., 2017a,c). Briefly, SOD activity was measured with a cytochrome *c* reduction inhibition reaction using the xanthine–xanthine oxidase system, indicated by an absorption value at 550 nm. The rate of H_2_O_2_ decomposition per 1 mg protein was used to measure CAT activity. GPx activity was measured by determining the coupled oxidation rate of NADPH during glutathione reductase recycling of oxidized glutathione from GPx regulated reduction of *t*-butyl peroxide. GR activity was determined by monitoring the NADPH-dependent oxidation of GSSG via the change in absorption value at 340 nm. GST activity was measured by its conjugation with 3,4-dichloronitrobenzene. MDA concentration was determined via the reaction of MDA and 2-thiobarbituric acid. All these enzyme activities were measure at 25°C, a common temperature for measuring enzyme activity in reptiles.

The assays for renal GLO activity and AA concentration were as described in a previous study (Chen et al., 2015). Briefly, GLO activity was analyzed as the AA concentration synthesized per gram tissue per hour. Total AA concentration (includes ascorbate and dehydroascorbate) was measured by the HPLC method and with an AA standard (10–20 μg AA mL^-1^).

### Statistics

A hierarchical clustering algorithm was generated with all standardized data according to a Euclidean method. Gene expression data were standardized to the fold change and then mapped to the interval (-1, 1), wherein the downregulated genes have a negative value and the upregulated genes have a positive value, for clustering analysis. The clustering analysis and the heatmap were conducted with R v. 3.5.0 (Development Core Team, 2018). All data are presented as mean ± SE with *P* < 0.05 accepted as a significant difference. All data were checked for normality and homogeneity of variance. One-way analysis of variance (ANOVA) followed by a Duncan *post hoc* test was employed for between-group comparison if normality and homogeneity of variance were both appropriate; otherwise a Kruskal–Wallis test followed by Mann–Whiney *U* *post hoc* test were used (Dytham, 2011). ANOVA analysis was conducted using SPSS package (v. 19, SPSS Inc., United States) and all graphs were made using Origin software (v. 9.0, OriginLab Corporation, United States) except the heatmap.

## Results

**FIGURE 1:**
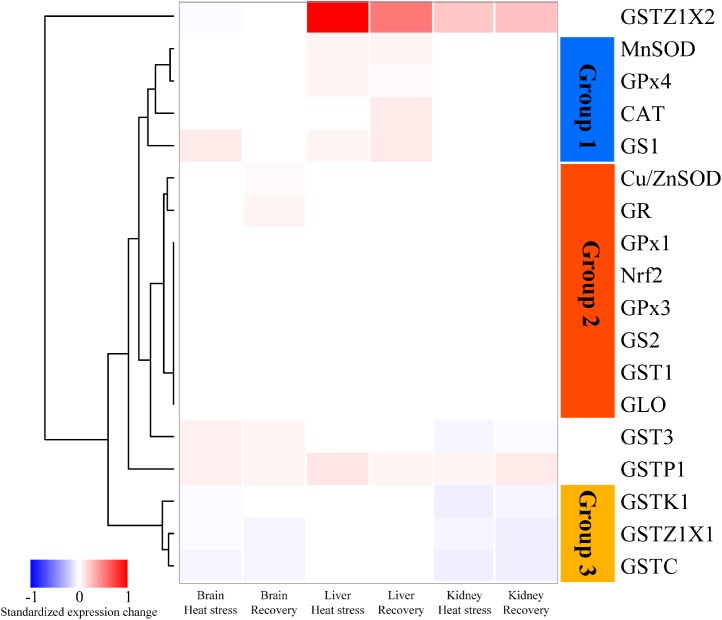
Heatmap clustering of antioxidant gene expression in brain, liver, and kidney of *P. sinensis*. The color scale indicates the standardized expression change in response to heat stress and recovery. The red indicated the upregulated value and the blue indicated the downregulated one. GLO only showed the data in the kidney because it only works specifically in the kidney.

Figure 1 shows a heatmap illustrating the relative changes in expression patterns by Nrf2 and multiple antioxidant enzymes measured in brain, liver and kidney of turtles exposed to heat stress (12 h at 33°C) and recovery (24 h back at 18°C), as compared with controls (18°C acclimated). The data clustered into three groups. In Group 1 (blue bar), genes including MnSOD, CAT and GPx4, mainly showed significant upregulation of expression in response to acute heat stress and/or recovery in the liver (Figure 1). Group 2 genes (orange bar) include Nrf2 and several antioxidant genes (Cu/ZnSOD, GPx1, GPx3, GS2, GR, GST1, and GLO). These showed relatively conservative gene expression responses (minor or not significantly different) to acute stress and subsequent recovery (Figure 1). Group 3 showed genes whose expression was mainly depressed by acute heat stress and following recovery in brain or kidney; these include three GST isoforms (Figure 1). In addition, three GST isoforms, GSTZ1X2, GST3, and GSTP1, also showed unique patterns of change (Figure 1).

**FIGURE 2:**
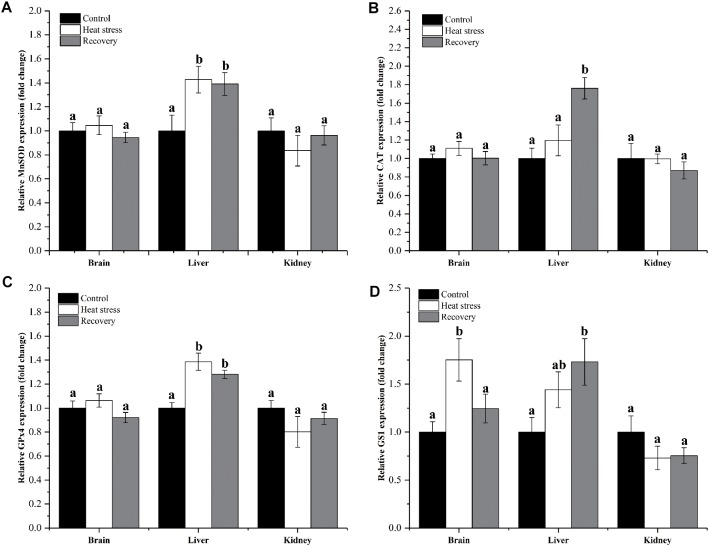
Expression change of genes in group 1. Data are presented as mean ± SE. Within each set of bars, superscripts without common letters indicate significant differences (*P* < 0.05). Panels are: **(A)** MnSOD, **(B)** CAT, **(C)** GPx4, and **(D)** GS1.

Expression changes of genes clustered in group 1 are shown in Figure 2. MnSOD and GPx4 mRNAs increased significantly by about 1.4-fold during acute heat stress and recovery in the liver, but not in the other two tissues (MnSOD: *P*_brain_ = 0.551, *P*_liver_ = 0.025, and *P*_kidney_ = 0.534; GPx4: *P*_brain_ = 0.184, *P*_liver_ < 0.001, and *P*_kidney_ = 0.297; Figure 2A,C). CAT mRNA transcripts increased only during the recovery period in the liver, by approximately 1.8-fold (*P*_brain_ = 0.371, *P*_liver_ = 0.002, and *P*_kidney_ = 0.527; Figure 2B). Expression of GS1 increased during acute heat stress in the brain and during the recovery period in the liver (both ∼1.75-fold) (*P*_brain_ = 0.014, *P*_liver_ = 0.044, and *P*_kidney_ = 0.281; Figure 2D).

**FIGURE 3:**
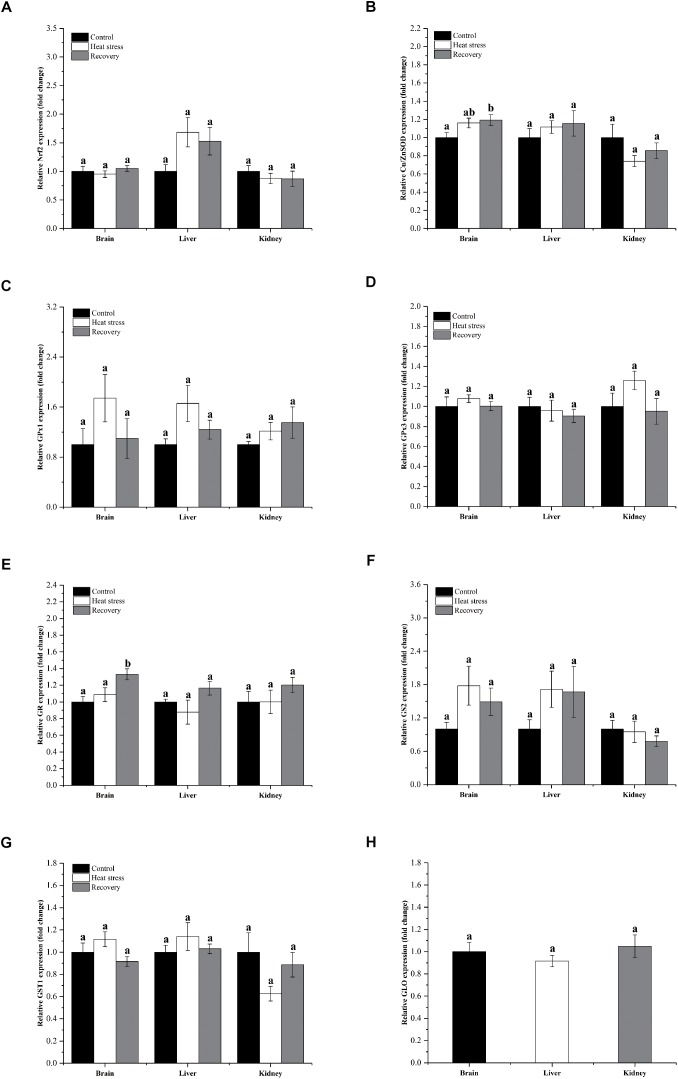
Relatively conserved gene expression in group 1. Data are presented as mean ± SE. Within each set of bars, superscripts without common letters indicate significant differences (*P* < 0.05). Panels are: **(A)** Nrf2, **(B)** Cu/ZnSOD, **(C)** GPx1, **(D)** GPx3, **(E)** GR, **(F)** GS2, **(G)** GST1, and **(H)** GLO.

Figure 3 shows detailed expression changes of genes clustered to group 2. Transcript levels of all these genes remained stable during acute heat stress and recovery in all three tissues except for Cu/ZnSOD and GR (all *P* values are given in Supplementary Table S1). Both Cu/ZnSOD and GR mRNA levels increased significantly during recovery in the brain of *P. sinensis* (Cu/ZnSOD: *P*_brain_ = 0.046, GR: *P*_brain_ = 0.011; Figure 2B,F). In addition, GLO expression in kidney did not change over the experimental course, and can also be classified into group 2 (*P* = 0.725; Figure 3H).

**FIGURE 4:**
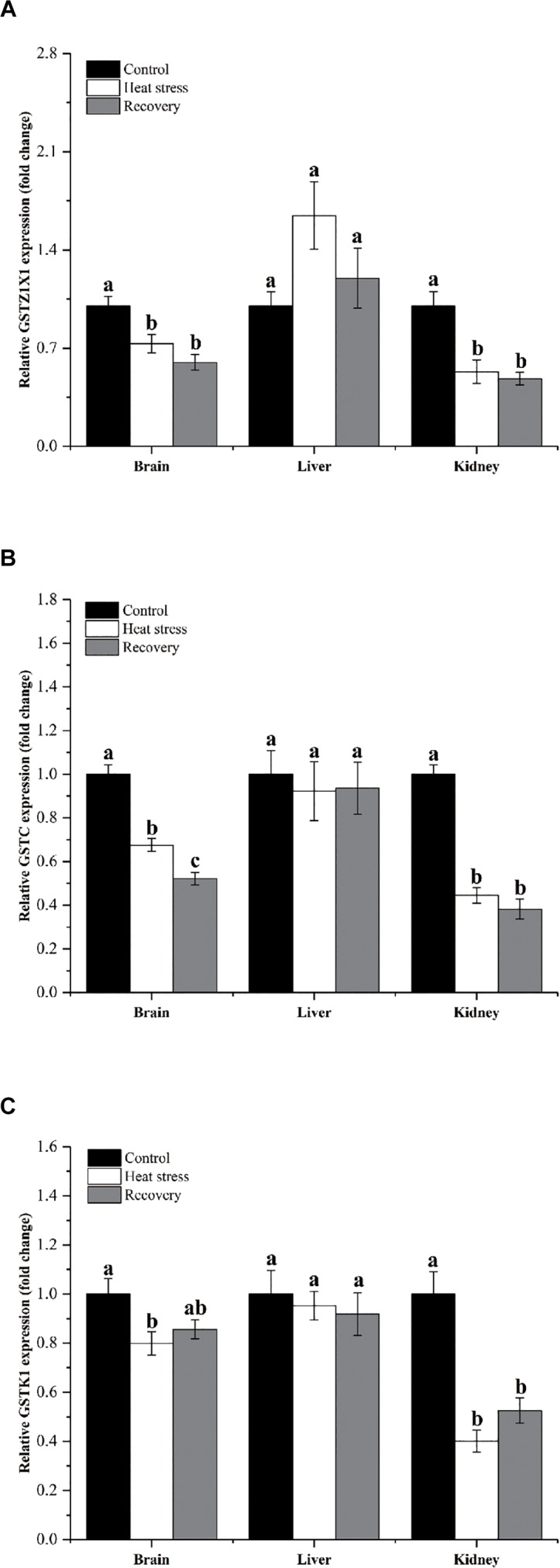
Expression change of genes in group 3. Data are presented as mean ± SE. Within each set of bars, superscripts without common letters indicated significant difference (*P* < 0.05). Panels are: **(A)** GSTZ1X1, **(B)** GSTC, and **(C)** GSTK1.

Figure 4 shows the expression changes of three GST isoforms. GSTZ1X1 mRNA decreased during acute heat stress and following recovery in the brain and kidney, but not in the liver (*P*_brain_ = 0.001, *P*_liver_ = 0.071, and *P*_kidney_ = 0.006; Figure 4A). GSTC showed the same pattern in all three tissues (*P*_brain_ < 0.001, *P*_liver_ = 0.891, and *P*_kidney_ < 0.001; Figure 4B). GSTK1 mRNA decreased by about 20% during acute heat stress in the brain and was reduced by 50–60% during both acute heat stress and following recovery in the kidney (*P*_brain_ = 0.029, *P*_liver_ = 0.777, and *P*_kidney_ < 0.001; Figure 4C).

**FIGURE 5:**
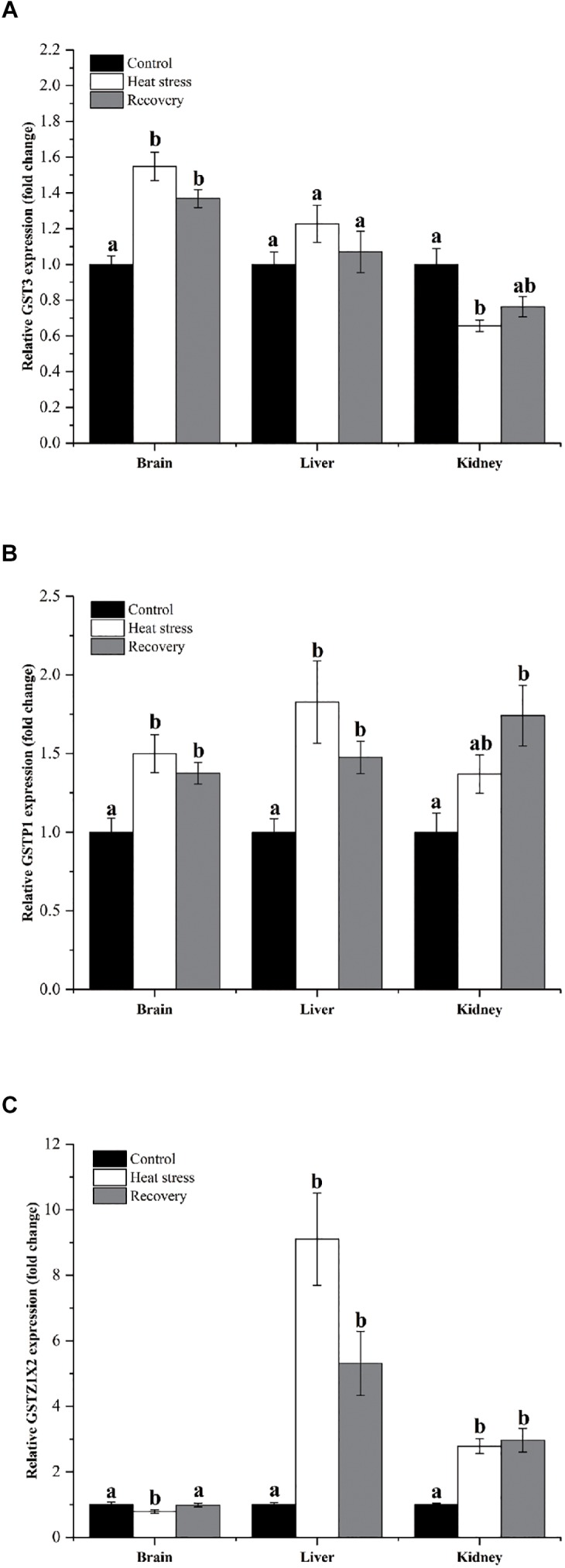
Expression changes by three GST isoforms with unique patterns. Data are presented as mean ± SE. Within each set of bars, superscripts without common letters indicated significant differences (*P* < 0.05). Panels are: **(A)** GST3, **(B)** GSTP1, and **(C)** GSTZ1X2.

Expression changes of three other GSH isoforms showed unique patterns of change, as shown in Figure 5. GST3 mRNA increased by about 1.4- to 1.5-fold during acute heat stress and recovery in the brain, but decreased during acute heat stress in the kidney (*P*_brain_ < 0.001, *P*_liver_ = 0.247, and *P*_kidney_ = 0.007; Figure 5A). GSTP1 showed increased mRNA during acute heat stress and recovery in the brain (1.5-fold) and liver (1.8-fold), whereas transcript levels increased significantly in kidney (1.70-fold) during the recovery period (*P*_brain_ = 0.004, *P*_liver_ = 0.003, and *P*_kidney_ = 0.008; Figure 5B). GSTZ1X2 expression was depressed during acute heat stress in the brain but was strongly elevated during both stress and recovery in the liver (nine and fivefold) and kidney (∼threefold) (*P*_brain_ = 0.048, *P*_liver_ < 0.001, and *P*_kidney_ = 0.001; Figure 5C).

**FIGURE 6:**
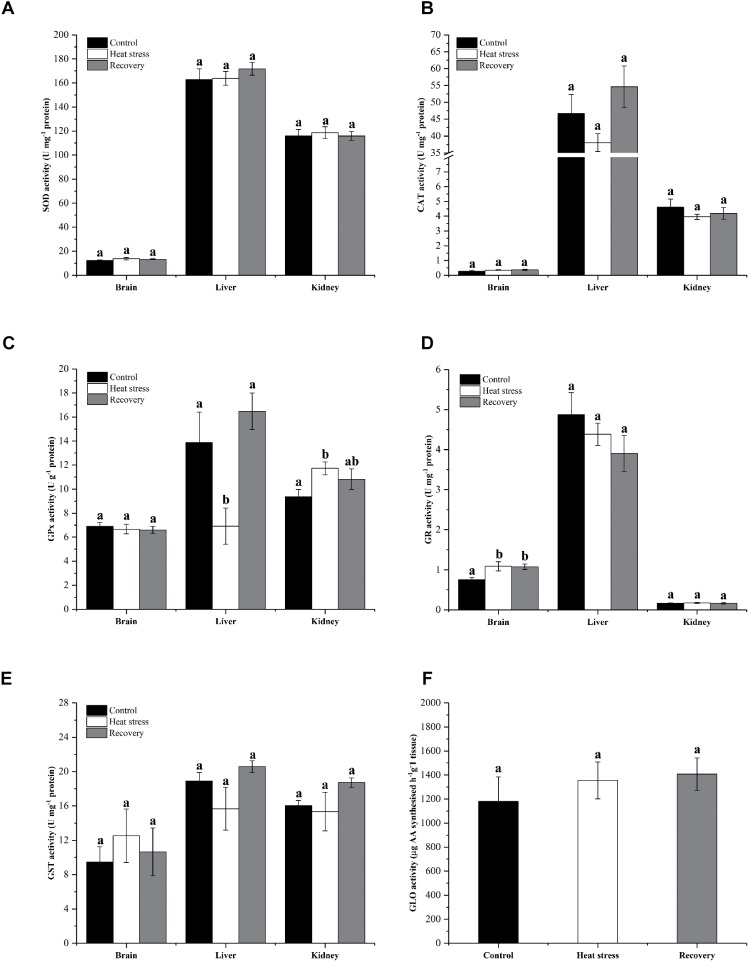
Activities of antioxidant enzymes. Data are presented as mean ± SE. Within each set of bars, superscripts without common letters indicated significant difference (*P* < 0.05). Panels are **(A)** SOD activity (U mg^-1^ protein), **(B)** CAT activity (U mg^-1^ protein), **(C)** GPx activity (U g^-1^ protein), **(D)** GR activity (U mg^-1^ protein), **(E)** GST activity (U mg^-1^ protein), and **(F)** nephric GLO activity (μg AA synthesized h^-1^ g^-1^ tissue).

Activities of six antioxidant enzymes are shown in Figure 6. Except for GPx and GR, enzyme activities did not change throughout the experiment in any tissue (all *P* values in Supplementary Table S1). Hepatic GPx activity decreased by ∼50% during acute heat stress but renal GPx activity increased ∼25% in the same period (*P*_brain_ = 0.796, *P*_liver_ = 0.010, and *P*_kidney_ = 0.04; Figure 6C). GR activity increased in the brain during acute heat stress and following recovery by ∼45% (*P*_brain_ = 0.021, *P*_liver_ = 0.330, and *P*_kidney_ = 0.934; Figure 6D).

**FIGURE 7:**
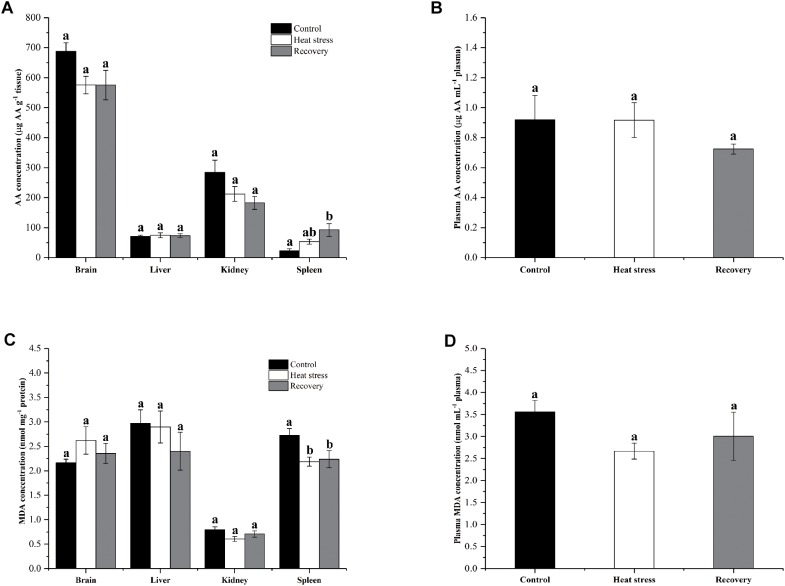
Change in AA and MDA concentrations in tissues and plasma of *P. sinensis*. Data are presented as mean ± SE. Within each set of bars, superscripts without common letters indicated significant difference (*P* < 0.05). Panels are **(A)** AA concentrations in tissues (μg AA g^-1^ tissue), **(B)** AA concentration in plasma (μg AA g^-1^ mL^-1^ plasma), **(C)** MDA concentration in tissues (nmol mg^-1^ protein), and **(D)** MDA concentration in plasma (nmol mL^-1^ plasma).

Figure 7 shows the changes in AA and MDA concentrations in four tissues and plasma of turtles. AA concentrations in the spleen significantly increased (fourfold over controls) during the recovery period (*P* = 0.011; Figure 7A). However, AA concentrations did not change significantly in other tissues during stress or recovery (*P*_brain_ = 0.082, *P*_liver_ = 0.903, and *P*_kidney_ = 0.06; Figure 7A) or in plasma (*P*_plasma_ = 0.376; Figure 7B). MDA concentration decreased by about 20% in the spleen of *P. sinensis* during both acute heat stress and recovery periods (*P* = 0.022; Figure 7C). In other tissues and plasma, MDA concentration did not change (*P*_brain_ = 0.324, *P*_liver_ = 0.437, *P*_kidney_ = 0.104, and *P*_plasma_ = 0.135; Figure 7C,D).

## Discussion

Acute heat stress usually links with intensely elevated metabolic rate in ectotherms, leading to greater oxygen consumption as well as increased ROS production. Enhanced antioxidant defenses induced by acute heat stress have been documented in several ectotherm groups, including snails, fish, and insects (Lushchak and Bagnyukova, 2006; Yang et al., 2010; Troschinski et al., 2014). In the present study, *P. sinensis* showed a relatively conservative antioxidant response to acute and intense heat stress at 33°C followed by 24 h recovery, which suggests a well-balanced performance to an extreme environmental stress.

Activation of Nrf2 expression has been observed in other animal species exposed to heat stress (Wang et al., 2015; Zachut et al., 2017). However, the current data for *P. sinensis*, indicates that the activation of Nrf2 at the transcript level may be unnecessary to the antioxidant response to acute heat stress. Our previous study found that expression of Nrf2 can be activated during the arousal (rewarming) period of hibernating *P. sinensis* hatchlings (Zhang et al., 2017a). It seemed that, after substantial development and growth, the constitutive expression of Nrf2 in the juvenile *P. sinensis* used in the present study appeared to be sufficient to mediate the response to temperature elevation without the need for further gene up-regulation, indicating a conservative response to acute heat stress in *P. sinensis* juveniles. In addition, an alternative yet non-conclusive explanation could be that other mechanisms enable longer half-life of Nrf2 protein in the cell/nucleus may also involve during the process, hence render an increase in Nrf2 mRNA expression is unneeded (Nguyen et al., 2009).

Antioxidant enzymes, including SOD, CAT, and GPx, make up the most important line of defense against oxidative stress in tissues. SOD reduces superoxide radicals to form hydrogen peroxide and both CAT and GPx function in clearing hydrogen peroxide (or other peroxyradicals) (Hermes-Lima and Zenteno-Savín, 2002; Ishii et al., 2002). In the present study, the slight increase in Cu/ZnSOD mRNA in the brain of *P. sinensis* during the recovery period indicated that there may be an increased oxidative stress at this time that triggered increased SOD expression. However, in the liver of *P. sinensis*, our results showed enhanced expression of several antioxidant enzymes during heat stress or following recovery, including MnSOD, CAT, and GPx4. MnSOD is an important isoform of SOD located in the mitochondria and its up-regulation indicates a potential oxidative stress in these organelles during acute heat stress (Jin et al., 2005). Such oxidative stress may be derived from increased superoxide production caused by an enhanced mitochondrial respiratory rate accompanying the rising metabolic rate that results from the elevated ambient temperature. In turn, oxidative stress may damage cell membrane lipids and stimulate increased GPx4 gene expression, whose protein product is known to provide special protection for organelle membrane systems (Brigeliusflohé and Maiorino, 2013). These effects lasted to the recovery period wherein an up-regulation of CAT expression was observed. Interestingly, our previous study found that increased CAT expression occurred in the liver of *P. sinensis* during the recovery period from acute cold stress (Chen et al., 2019). This indicates that activation of CAT is important for *P. sinensis* during the recovery process from intense temperature fluctuation although it did not appear to be sensitive to the temperature stress itself. It is also worth noting that the isoform of SOD or GPx which responded to acute heat stress showed lower constitutive expression levels in the tissues of *P. sinensis* than other isoforms, which was also observed in our previous studies (Zhang et al., 2017a). This may indicate that *P. sinensis* regulates isoforms of antioxidant enzyme genes with normally low expression levels while maintaining those with more dominant expression at stable levels in response to acute heat stress or recovery. We also observed that the changes in antioxidant gene expression did not result in a corresponding change of enzyme activities in tissues of *P. sinensis*, which may due to post-transcriptional regulation and was also reported in our previous studies (Zhang et al., 2017a; Chen et al., 2019). In other animals, increased activities of SOD or CAT were observed during heat stress. For example, heat stress (Δ14°C for 12 h) increased SOD activity by fourfold in the brain and liver in goldfish *Carassius auratus* (Lushchak and Bagnyukova, 2006). Similarly, heat stress (Δ6°C for 12 h) induced increased CAT activity in the fish *Onychostoma macrolepis* (Yu et al., 2017). Considering the intensity of the acute heat stress used in the present study (Δ15°C in less than 5 min and maintained for 12 h), these stable enzyme activities in tissues of *P. sinensis* may indicate a conservative antioxidant enzyme response to acute heat stress or recovery. Analysis of GPx activity, however, showed a different response that compared with the other two antioxidant enzymes. We propose that the decreased in hepatic GPx activity may due to a reduced need for GPx because it shares a similar antioxidant function with CAT. In addition, since GPx needs GSH as a substrate, its activity might be limited if thermal stress impaired glutathione metabolism. In the kidney, the increased GPx activity during heat stress may be related to enhanced metabolic capacity with rising temperature. We have reported that nephric GPx activity can be depressed by acute cold stress in *P. sinensis* (Chen et al., 2019), and thus, nephric GPx activity of *P. sinensis* is more sensitive to temperature fluctuations than other enzymes.

Glutathione and AA are important antioxidant scavengers in animals, especially in freshwater turtles (Chen et al., 2015; Zhang et al., 2017c). GSH works as the essential substrate of GPx or GST and is synthesized *de novo* by GS or replenished to its reduced form by GR (Saez et al., 1990). It was noteworthy that a potential need for greater GSH content can be inferred in the brain of *P. sinensis* from enhanced GS1 expression and GR activity during acute heat stress as well as increased transcription and activity levels of GR during recovery. This may help to maintain the redox balance in brain of *P. sinensis* during acute heat stress and recovery. By contrast, in the liver, our treatments mainly affected the expression of GS1, an GS isoform with relatively lower constitutive expression levels than GS2 in tissues of *P. sinensis* (Zhang et al., 2017c). In the GSH system, the most impressive change in pattern was observed for GST isoforms, an important family of detoxifying enzymes. Since GSTP1 was highly inducible by acute heat stress and recovery in all three tissues, it may prove be useful as a biomarker of heat stress in *P. sinensis*. The expression levels of different GST isoforms may compensate for each other to some extent, which then resulted in an overall stable GST activity in tissues of *P. sinensis*. Similar results were observed in hibernating *P. sinensis* hatchlings (Zhang et al., 2017c). It seems that net GST activity is stable in the stress response of *P. sinensis* despite of fluctuations in transcript levels of its different isozymes.

Acute heat stress and recovery caused increased splenic AA concentration along with decreased MDA levels, which was also observed in kidney of *P. sinensis* in arousal (rewarming) from hibernation (Chen et al., 2018). Both of these results indicate an essential protective role of AA for *P. sinensis* tissues, especially during the recovery period from stress. Meanwhile, our previous study also indicated that spleen is sensitive to intense or prolonged oxidative stress (Zhang et al., 2019). Thus, we proposed that the spleen requires extra antioxidant protection, in the form of increased AA concentrations, during acute heat stress and recovery. This may be of the results of limited AA storage capacity of this small-sized tissue or dealing with the needs of the hematological function of spleen, which could be important in meeting metabolic demands during acute heat stress. When AA replenishment is limited or cannot meet the needs of the spleen under stress conditions, *P. sinensis* may be at risk of oxidative damage to the spleen (Chen et al., 2018). Therefore, one key adjustment needed for *P. sinensis* to endure acute heat stress or frequent extreme weather may be to maintain an effective and sufficient AA supply for important tissues, such as spleen.

From a systematic view, our results suggest a relatively conservative antioxidant response strategy and also a good tolerance of acute heat stress of *P*. *sinensis*. In this response strategy, the turtle maintains high constitutive expression and activity levels of most antioxidant enzymes in important tissues when being exposed to environmental stress. Meanwhile, in the liver, the most important metabolic tissue, the isoforms of antioxidant enzymes with relatively lower constitutive expression levels responded to temperature elevation. Another important process is the rapid regulation of AA storage in tissues (especially spleen) to meet the extra need of antioxidant defense. Interestingly, a similar strategy was also found in *P. sinensis* dealing with cold stress and recovery (Chen et al., 2019). Together these two studies indicate a good adaptive capacity of the antioxidant defense system in this subtropical freshwater turtle to acute temperature fluctuation. The finding that these turtles rely on stable/conservative and basal constitutive antioxidant defenses to endure environmental stress, i.e., acute heat stress in this case, was also congruent with the strategy identified in similar studies on other turtle species under various stress contexts (Willmore and Storey, 1997a; Baker et al., 2007). These all suggested a unique but shared stress response strategy in turtles as comparing with other animals. The stable and conservative antioxidant response allows *P. sinensis* to endure the heat stress employed here but it also suggested that this conservative antioxidant response may not always be able to meet the need for antioxidant protection under more severe or prolonged stress conditions. In particular, the AA replenishment capacity in the spleen may affect the destiny of *P. sinensis* during acute heat stress, in the content of more frequent extreme hot weather.

In conclusion, this is the first study to assess the antioxidant response to acute heat stress in a freshwater turtle species. A novel systemic antioxidant response was observed in tissues of *P. sinensis* that was different from those seen in other animal groups. We can summarize three main changing patterns of antioxidant genes in *P. sinensis* during acute heat stress and recovery: (1) Acute heat stress or recovery caused increased gene expression of several isoforms of antioxidant enzyme genes in the liver of *P. sinensis*; notably, these were isozymes that showed relatively low constitutive expression levels as compared with than other isoforms in the same gene family; (2) Expression of most antioxidant genes and Nrf2 did not change in brain, liver and kidney of *P. sinensis* throughout; (3) Acute heat stress and recovery affected the expression of GST genes in the brain and kidney of *P. sinensis*. Overall, antioxidant enzyme activities remained stable and no oxidative damage was observed, as indicated by MDA levels, in tissues of *P. sinensis.* The data also indicate that the spleen needs extra protection from AA to endure acute heat stress and recovery periods. Our study revealed a relatively conservative antioxidant defense system, which can allow *P. sinensis* to endure intense temperature elevation, from 18 to 33°C with no oxidative damage.

## Ethics Statement

The experiments were conducted according to the standards of the Ethics and Animal Welfare Committee (EAWC) of Beijing Normal University (Approval No. CLS-EAW-2014-010).

## Author Contributions

BC and CN conceived the study. WZ, BC, LY, and HJ collected the data. WZ and BC analyzed the data. WZ wrote the initial draft of the manuscript. BC, CN, and KS revised the manuscript.

## Conflict of Interest Statement

The authors declare that the research was conducted in the absence of any commercial or financial relationships that could be construed as a potential conflict of interest.

## Supplementary Material

TABLE S1 *P* values and statistics for all parameters in tissues of *P. sinensis* during acute heat stress and recovery periods.
